# Viewpoint on the Consequences and Mitigation of Cognitive Bias in the Radiological Interpretation of Breast Cancer Imaging Using Artificial Intelligence

**DOI:** 10.2196/78955

**Published:** 2026-03-30

**Authors:** Lorenzo Conti, Benedetta Capetti, Ottavia Battaglia, Roberto Grasso, Filippo Pesapane, Dario Monzani, Gabriella Pravettoni

**Affiliations:** 1 Applied Research Division for Cognitive and Psychological Science, European Institute of Oncology IRCCS, Via Ripamonti 435, Milan, 20141, Italy, +39 0294371083; 2 Department of Oncology and Haemato-Oncology, University of Milan, Milan, Italy; 3 Breast Imaging Division, European Institute of Oncology IRCCS, Milan, Italy; 4 Department of Psychology, Educational Science and Human Movement, University of Palermo, Palermo, Italy; 5 WeSearch Lab – Laboratory of Behavioral Observation and Research on Human Development, University of Palermo, Palermo, Italy

**Keywords:** radiological assessment, artificial intelligence, AI, cognitive bias, breast cancer, imaging

## Abstract

Artificial intelligence (AI) is increasingly integrated into breast imaging workflows, offering the potential to enhance diagnostic accuracy, efficiency, and early cancer detection. Image interpretation plays a pivotal role in the breast cancer diagnostic pathway, directly influencing therapeutic decisions and patient outcomes. However, the effective implementation of AI-assisted systems relies not only on technical performance but also on radiologists’ trust, acceptance, and readiness to incorporate these tools into clinical practice. In addition, system-related, perceptual, and cognitive factors may contribute to diagnostic errors, ultimately affecting overall accuracy and reliability. This paper provides a comprehensive overview of the cognitive and systemic sources of diagnostic inaccuracies in breast imaging, emphasizing the growing role of AI as both a supportive and potentially bias-modulating tool. Recent prospective studies have demonstrated the clinical safety and effectiveness of AI-assisted mammography screening, reporting improved cancer detection rates and reduced workload. Nonetheless, the integration of AI into diagnostic workflows without an appropriate knowledge of the consequences may introduce new cognitive biases, such as anchoring, automation, and confirmation bias, that influence radiologists’ decision-making and counteract the intended benefits. To address these challenges, the paper outlines strategies to mitigate diagnostic errors and foster appropriate integration of AI into clinical practice. These include targeted training programs, enhanced interdisciplinary communication, and standardized interpretation workflows that promote consistent evidence-based practice. Furthermore, the adoption of explainable AI approaches is identified as a key factor in improving model transparency and interpretability, allowing radiologists to understand algorithmic reasoning and engage in a more informed, confidence-based human-AI collaboration. Ultimately, a balanced and context-sensitive integration of AI, grounded in continuous professional education and cognitive awareness, is essential for improving diagnostic accuracy while preserving radiologists’ critical analytical skills.

## Radiological Interpretation Workflow

The increasing integration of artificial intelligence (AI) into clinical medicine has created new opportunities to enhance diagnostic accuracy and therapeutic decision-making across multiple fields. AI is defined as a machine’s capability to mimic intelligent human behavior, such as learning, reasoning, and problem-solving [1]. Despite its promise, AI adoption in routine clinical practice faces significant challenges, including disparities in medical imaging access, and remains in early stages across many specialties [2-4].

In oncology, AI facilitates early tumor detection and personalized treatment planning, with a prominent role in radiology, where it analyzes medical images and patient data to detect patterns and abnormalities, accelerating and improving diagnosis. However, its extensive use in clinical decision-making increases exposure to bias with potentially serious clinical repercussions [2].

Radiological interpretations critically influence therapeutic decisions and patient management outcomes [5,6]. These interpretations are based on the careful evaluation of available evidence but remain subject to limitations because of the subjective nature of image assessment, without replacing gold-standard pathological examinations [5]. In health care, AI has been introduced to support complex tasks that require reasoning and decision-making [7]. However, its application must be approached cautiously, as it does not eliminate the possibility of errors.

Given these limitations, it is essential to understand the factors contributing to diagnostic variability and potential errors in radiology.

The process of interpreting medical images is complex, requiring radiologists to evaluate imaging data meticulously to identify potential health issues. Despite their expertise, the diagnostic error rate among practicing radiologists is estimated to be between 3% and 6%, amounting to approximately 40 million errors globally each year, given annual imaging volumes [8,9]. This error rate is even higher, between 31% and 37%, in cross-sectional imaging such as computed tomography (CT) [10]. Given the growing reliance on cross-sectional imaging, it is essential to understand the causes, prevalence, and impact of diagnostic errors to minimize their effect on patient care.

Diagnostic errors have significant consequences for patient outcomes and place a substantial burden on health care systems. They can be categorized into 3 primary groups: system errors, visual-perception errors, and cognitive errors [4,11]. System-related errors include equipment failures or suboptimal image quality. Visual-perception errors occur when radiologists either fail to detect abnormalities or mistakenly classify them as normal, influenced by factors such as fixation duration [5]. Finally, cognitive errors result from incorrect interpretation of detected abnormalities and represent the largest proportion of diagnostic mistakes, reaching up to 74% in some imaging modalities [12].

This paper aims to provide an in-depth overview of the implementation of AI in medical imaging, with a specific focus on breast cancer radiology, based on a comprehensive literature search (see Multimedia Appendix 1). This focus distinguishes our work from previous reviews, such as that of Stogiannos and colleagues [4], which adopt a modality-independent perspective. In contrast, the present review concentrates on how AI is being integrated into real-world breast imaging workflows across screening, diagnostic, and interventional settings. Particular attention is given to the practical challenges of implementation and the evolving dynamics of human-AI interaction in daily clinical practice.

Adopting both a conceptual and practice-oriented lens, it explores the dual nature of AI as both a supportive adjunct and a potential source of diagnostic error, emphasizing strategies for informed and responsible AI use. Finally, it addresses cognitive biases that arise from human-AI collaboration, their implications for clinical decision-making, and possible approaches to mitigate these effects.

Through this perspective, we argue that understanding and managing these biases is crucial to ensuring the safe and effective integration of AI in breast imaging, with the ultimate goal of improving diagnostic accuracy and optimizing patient care.

## The Adoption of AI in Radiological Senology

Breast cancer is one of the most diagnosed cancers worldwide, accounting for approximately 2.3 million new cases and 0.7 million deaths among women each year [13]. Early diagnosis is of pivotal importance in reducing morbidity and mortality, and, in this scenario, mammographic screening plays a crucial role, currently being the only screening test that has been shown to reduce breast cancer–related mortality [14].

Despite the proven efficacy of mammographic screening, several challenges persist, particularly in terms of workload and diagnostic consistency. Screening programs are typically addressed to women aged between 40/50 and 70/80 years, with European guidelines recommending double reading of screening mammograms to ensure high sensitivity [15]. Nevertheless, the shortage of experienced breast radiologists and the large workload render the double reading difficult to sustain in several countries [16].

A possible solution might be represented by image analysis tools based on AI, a field of computer science dedicated to the creation of systems performing tasks that usually require human intelligence, aimed, for example, at facilitating triage of screening examinations according to risk of malignancy or supporting the radiologist’s decision with computer-aided detection (CAD) highlighting suspicious findings, ultimately reducing the workload and the number of interval cancers [17].

Retrospective studies [18-20] suggest that the accuracy of AI is similar to or better than that of breast radiologists and that AI could help radiologists reduce false negative screening results when used as detection support, as it has been shown that AI retrospectively classifies screening examinations as high-risk before a diagnosis of interval cancer [21-23].

A breakthrough point was reached in 2023, when Lång et al [24] published the results of their randomized, controlled mammography screening with artificial intelligence (MASAI) trial, a study investigating an AI-supported screen-reading procedure involving triage of screening examinations to single or double reading, along with detection support, compared to standard double reading.

The MASAI trial was designed as a randomized, parallel, noninferiority, single-blinded, controlled, screening accuracy study in which, after screening, mammograms were acquired, and examinations were automatically randomized to AI-supported screening (intervention group) or standard double reading without AI (control group).

The examinations randomized to the intervention group were analyzed using Transpara version 1.7.0 (ScreenPoint Medical), a deep learning system to identify and interpret mammographic regions suspicious for cancer. The AI system provided an examination-based malignancy-risk score on a continuous scale ranging from 1 to 10: examinations were considered as low risk (risk scores 1‐7), intermediate risk (risk scores 8 and 9), or high risk (risk score 10). Furthermore, the AI system provided CAD marks at suspicious regional findings [24].

In the intervention group, examinations with risk scores of 1‐9 (low and intermediate risk) underwent single reading, and examinations with risk scores of 10 (high risk) underwent double reading, done by two different breast radiologists [24].

Readers first read the examination without CAD marks and then with CAD marks, if available.

In the control group, screening examinations were read with standard unblinded double reading without AI.

This clinical safety analysis testifies that a screen-reading procedure using an AI tool to triage screening examinations to single or double reading and with the use of AI as detection support in mammography screening was safe because the cancer detection rate (6.1 per 1000 participants screened) was above the prespecified lower limit for safety and was similar to that of double reading without AI (5.1 per 1000). The use of AI did not influence the rates of recalls, false positives, or consensus meetings, while the screen-reading workload was reduced by almost half. Indeed, this study found that the benefit of AI-supported screening regarding screen-reading workload reduction was considerable.

Furthermore, the results of the trial showed that AI-supported screening detected 20% more cancers (244 vs 203) compared to standard screening and increased detection of in situ cancers with AI-supported screening compared with standard screening (60 vs 38), which could be concerning in terms of overdiagnosis.

Nevertheless, improvement in one performance measure, such as cancer detection rates, does not always imply an improvement in terms of outcome, such as recurrence rates or mortality reductions. The results of future screening cycles are going to highlight whether the introduction of new strategies, such as AI-supported screening, has a positive impact on the overall effect of the intervention on outcomes and if the costs are justified [25].

The results of this prospective randomized trial corroborate the findings of several retrospective studies that have shown AI has sufficient diagnostic accuracy to make radiological reads as an independent reader of screening mammograms. Nevertheless, there is currently an absence of specific guidelines regarding the potential introduction of AI as an independent reader or, more broadly, for the application of AI tools in everyday clinical practice [26,27].

A comprehensive systematic review performed by Anderson and collaborators [28], focused on the external validation of AI technologies applied to mammographic screening in real-world clinical settings, highlighted that the majority of studies evaluating either stand-alone AI systems or AI used in conjunction with radiologists reported improved diagnostic accuracy compared to radiologist-only interpretations. Some studies included in the review indicate that combining outputs from several AI models—ensemble modeling—can enhance overall performance compared to relying on a single algorithm [29,30]. However, implementing such approaches in real-world clinical environments may be challenging, particularly when it involves integrating multiple AI system owners. Concerning prospects, they emphasized a significant gap in external validation research, which is essential to assess how well AI models perform across large and varied populations in terms of different race/ethnicities or breast cancer risk profiles, and on different imaging techniques such as digital breast tomosynthesis (DBT) versus digital mammography. Additionally, it was highlighted that there is a need to determine the specific screening scenarios, such as age groups and screening intervals, where AI applications would be most beneficial.

Similarly, Houssami and collaborators [31] reported a satisfying diagnostic accuracy for the AI models proposed by the studies involved in their scoping review, with a median area under the curve of 88.2%. What was pointed out in their work was that most of the existing AI research in breast imaging has relied on data from screen-film or digital mammography. Given that DBT is increasingly becoming a central character in breast cancer screening, future AI development should incorporate DBT imaging data, as AI tools trained solely on traditional 2D mammography may quickly become obsolete as DBT adoption grows in clinical practice [31].

## Diagnostic Errors in Radiology

Due to the complex interpretational process executed by radiologists, it may be subject to diagnostic errors in up to 6% of cases [5,6]. Diagnostic errors include system errors, such as those related to the equipment used (ie, poor image quality or suboptimal technique), and visual-perceptual errors, which occur when clinically significant findings are not identified due to specific risk factors, including reader fatigue, rapid interpretation, distractions, or interruptions [5]. However, there is a third type of error, called “cognitive errors,” related to the clinicians’ judgment, when clinically significant findings are correctly identified, but an incorrect interpretation is attributed due to mental shortcuts or preconceptions [32]. Identifying these errors is crucial in the medical field, as it enables early action to prevent inaccurate interpretations.

This type of error is caused by the influence of cognitive biases, systematic and unconscious thinking errors that occur when processing and interpreting information in the environment, and that influence people’s decisions and judgments [33,34].

Over time, various classifications have been proposed to categorize cognitive bias within the field of radiology, with multiple reviews documenting the main typologies encountered during medical image interpretation. These cognitive biases have been identified as significant contributors to radiological errors and diagnostic inaccuracies [5,35]. Furthermore, with the increasing integration of AI into radiological clinical practice, it becomes essential to consider its impact within the theoretical framework. In medical AI, a bias is defined as any factor or prejudice that drives an AI algorithm to generate differential or inequitable outcomes [2]. Consequently, the implementation of AI must be carefully evaluated, as it holds the potential to both mitigate certain diagnostic errors and exacerbate existing cognitive biases [8]. Indeed, AI has shown promise in reducing subjective prejudices, enhancing image analysis clarity, decreasing interobserver variability, and improving the speed and accuracy of radiological reporting [1]. However, AI algorithms trained on biased datasets may perpetuate or even amplify existing health disparities, leading to compromised diagnostic accuracy and inequities in patient treatment [36]. These issues introduce new challenges that health care professionals must acknowledge, particularly regarding the emergence and influence of cognitive biases in AI-augmented radiology [1].

## Overview of Cognitive Bias and AI-Driven Insights

This section describes the main cognitive biases highlighted in the literature regarding radiological interpretation and explores the potential contribution and pitfalls associated with AI application in this medical context. To facilitate a clear understanding and contextualization of these errors, Table 1 summarizes each cognitive bias with a specific clinical example that realistically illustrates its occurrence in practice.

**Table 1.** Overview of cognitive bias.

Cognitive bias	Explanation	Practical example	AI^a^ contribution
Influence of AI (evidence-based)			
Availability	Overemphasizes easily recalled cases over statistical prevalence	*A breast radiologist has recently dealt with several cases of aggressive triple-negative breast cancer in young patients. Shortly afterward, when performing a preventive check-up in a 30-year-old with vague symptoms and no risk factors, the radiologist overestimates the likelihood of malignancy and recommends an aggressive workup, despite weak evidence. The recent memory of similar high-impact cases is fresh and vivid (availability bias), so the radiologist gives disproportionate weight to that experience, leading to an overestimation of the probability of cancer in situations that statistically may not warrant it*.	AI shifts focus to statistical data rather than anecdotal memory [32]
Nonavailability	Ignores diagnoses not previously encountered	*A breast radiologist, during routine screening, fails to consider a rare presentation of breast cancer because they have never seen a similar case before. This is nonavailability bias, as the radiologist underestimates or overlooks the possibility of cancer because that diagnostic pattern isn’t readily available in their memory or experience*.	AI trained on large datasets can identify rare cases effectively [32]
Confirmation	Selectively interprets data to confirm existing hypothesis	*A general practitioner suspects a benign fibroadenoma in a patient with a palpable lump and refers the patient to a breast radiologist for an ultrasound. The radiologist identifies a round, circumscribed mass and, believing that this finding aligns with their first impression, the radiologist selectively focuses on features that support a benign interpretation and ignores subtle signs, like nonparallel orientation and heterogeneous echostructure, that could suggest a phyllodes tumor or malignancy. This is confirmation bias: the radiologist seeks out or gives more weight to evidence that supports an existing belief or hypothesis (in this case, that the mass is benign) while not giving enough importance to contradictory evidence, which can lead to diagnostic error or delayed treatment*.	AI may amplify bias if radiologists unquestioningly accept AI suggestions [37]
Anchoring	Fixates on initial information despite conflicting later evidence	*Example 1: A real-life scenario might be represented by a patient with a history of a benign breast cyst returning for follow-up imaging examinations. The breast radiologist identifies a similar-appearing mass in the same location and assumes it’s the same cyst, labeling it as benign without carefully evaluating prior images or subtle changes. Nevertheless, the lesion has grown and, in the present examination, new features of malignancy have appeared (eg, irregular margins and vascularization). The radiologist fixates on the initial impression or past diagnosis (a benign cyst) and fails to adequately update (anchoring bias) their judgment in light of new evidence.* *Example 2: A breast radiologist is reviewing a mammogram assisted by an AI tool that highlights a lesion and assigns a low malignancy score. Trusting the AI’s judgment, the radiologist anchors on this benign assessment and disregards their initial concern about subtle spiculations, ultimately reporting the lesion as likely benign. However, a biopsy performed later confirms that the lesion was an invasive carcinoma*.	AI may exacerbate if the radiologist overly trusts the AI’s initial assessment [37]
Automation	Prefers automated AI outputs even when conflicting info exists	*During a busy screening session, a radiologist uses an AI tool that flags suspicious lesions with heat maps on mammograms. One mammographic examination shows no AI-detected abnormalities, so the radiologist quickly signs the report as negative, without further close review of the mammograms. In this way, a small spiculated mass, missed by the AI tool goes undetected*.	Risk of overreliance on AI leading to missed diagnoses [4]
Framing	Interpretation influenced by how clinical info is presented	*A surgeon sends a patient complaining of a palpable lump to a breast radiologist for an ultrasound, highlighting the patient’s strong family history of breast cancer and assessing that, according to him, this finding is suspicious. The radiologist, influenced by this alarming clinical context, interprets a likely benign nodule as suspicious and recommends a biopsy, even though imaging features alone don’t strongly support malignancy*.	AI could reinforce framing if trained on biased clinical data [36]
Premature closure	Accepts the diagnosis too early without considering alternatives	*A breast radiologist identifies a well-circumscribed, round opacity on a mammogram and immediately labels it as a benign nodule (eg, possible expression of a fibroadenoma) based on appearance and the patient’s young age, without recommending further examinations; however, the mass was a rare presentation of a malignant phyllodes tumor. This is premature closure bias: the radiologist settles too quickly on a diagnosis without fully considering alternative possibilities or completing the necessary diagnostic workup, leading to missed or delayed diagnosis, especially in atypical cases.*	AI might help by suggesting alternative diagnoses for consideration [36]
Influence of AI (theoretical/hypothesis-driven)^b^			
Satisfaction of report	Accepts previous radiological reports without independent reassessment	*A patient is referred for a diagnostic mammogram after a recent ultrasound (done at another facility), reporting a suspicious lesion in the left breast at 2 o’clock. The breast radiologist, assisted by an AI system that flags suspicious regions, identifies on the mammogram, in the same location where the lesion was reported on ultrasound, a spiculated lesion with a high-risk score for malignancy assigned by the AI tool. The radiologist “satisfied” that the main finding had been identified, both by the AI and by their own review, and stopped further searching, and missed other suspicious lesions. Indeed, on the contralateral breast, there was a small cluster of irregular microcalcifications, suspicious for an in situ lesion*.	AI could provide independent second reads to limit this bias
Attribution	Preconceptions or patient stereotypes influence diagnosis, neglecting relevant info	*A breast radiologist is interpreting a screening mammogram of a woman with a history of prior false-positive findings (eg, cluster of microcalcifications that warranted a vacuum-assisted biopsy under stereotactic guidance, with an oncologically negative result) and assumes her current finding is, even this time, a benign one. This is an attribution bias as the radiologist allows prior patient characteristics or history to inappropriately influence interpretation. Instead of evaluating the image solely on current findings, the radiologist may attribute it to past behavior or outcomes, potentially leading to a missed diagnosis*.	AI may be reduced by focusing on current image data, not patient history
Satisfaction of search	Stops searching for additional abnormalities after finding one	*A breast radiologist identifies a clear spiculated mass in the left breast on a mammogram and correctly reports it as suspicious for malignancy. Satisfied with having found an obvious abnormality, the radiologist stops looking carefully and misses a second suspicious finding: a small cluster of microcalcifications in the contralateral breast*.	AI could help by prompting a full image review
Inattentional blindness	Attention fixation causes missed visible findings	*A breast radiologist is reviewing a mammogram that was previewed by a resident, who believes that there are suspicious microcalcifications on the right upper quadrant; this primes the attending physician to look for calcifications in that specific quadrant. While intensely focused on identifying calcification patterns in that area, the radiologist fails to notice an opacity in a different quadrant*.	AI may highlight all findings objectively, reducing this bias
Hindsight	Overestimates the predictability of diagnosis based on outcome knowledge	*A breast radiologist is reviewing a mammogram taken 12 months before a patient was diagnosed with breast cancer. Knowing the eventual diagnosis, the radiologist now clearly identifies the lesion: a small spiculated mass. Nevertheless, at the time of the original screening, the lesion was subtle, not clearly identifiable due to the high breast density, and not highly suspicious without the knowledge of the outcome*.	AI may provide consistent, unbiased analysis, unaffected by outcome knowledge
Regret	Overcompensation due to fear of past diagnostic miss	*A breast radiologist previously missed a case of subtle ductal carcinoma in situ (DCIS) that led to delayed diagnosis. For this reason, in subsequent cases, the radiologists begin to define benign-appearing calcifications as suspicious, overrecommending biopsies, driven by a fear of repeating the error. Emotional reactions to a past mistake influence the radiologist’s judgment: this is regret bias, and the desire to avoid the regret of another missed cancer leads to overcompensation, increasing unnecessary procedures and patient anxiety*.	AI could mitigate by providing probabilistic assessments, reducing emotion-driven bias
Commission	Taking an action without an objective basis for the course of action	*A breast radiologist, while analyzing a clinical mammography of an asymptomatic patient with no personal or family history of breast cancer, identifies a vague asymmetry in the outer quadrant of the left breast, only visible in one projection and basically unchanged in comparison to previous exams, thus likely representing a normal glandular area. Despite the report of this exam being concluded as negative/ BI-RADS 1, the radiologist decided to assign a BI-RADS 3 to the finding, recalling the patient for additional examinations that were all negative*.	AI could mitigate commission bias by acting as a second objective reader, limiting excessive subjective assessments of benign findings

a AI: artificial intelligence.

b For biases marked as “theoretical/hypothesis-driven,” current evidence is extrapolated from the broader literature on cognitive biases, AI-assisted decision-making, and diagnostic radiology. Direct empirical evidence specific to breast cancer imaging workflows is currently limited. Language such as “may,” “could,” and “theoretically” indicates effects that are conceptually plausible but await clinical validation.

One prominent cognitive bias is the availability bias, where radiologists may overemphasize experiences that are easily recalled to elaborate on the diagnosis, ignoring the base rate or prevalence rate of a particular disease [33,35,38,39]. Conversely, radiologists who have never experienced a specific symptom may exhibit a nonavailability bias, resulting in the oversight of potential diagnoses associated with that symptom [5].

AI could help to limit the availability bias, shifting the focus from anecdotal evidence to statistical patterns by processing comprehensive datasets that human cognition cannot effectively synthesize [32].

Similarly, the confirmation bias is another critical cognitive error where radiologists may selectively gather and interpret clinical data to reaffirm an existing hypothesis while disregarding alternative possibilities [5,35]. Also, in this context, AI may contribute to increasing the occurrence of this cognitive bias by encouraging radiologists to confirm AI-suggested diagnoses without adequately considering alternative possibilities [37]. Conversely, when AI is trained using controlled databases, it provides objective insights that are independent of human preconceptions, thereby reducing the frequency of this bias [32]. This bias is particularly concerning as it can significantly impair diagnostic accuracy.

An opposite example is provided by the anchoring bias that manifests when radiologists tend to fixate on preliminary information despite receiving subsequent data that conflicts with their initial diagnosis [5,33,38,40]. In this case, the use of AI could accentuate the probability of making errors, leading the radiologist to be influenced by the initial results provided by it [37]. This fixation can lead to a failure in adapting their assessment to new evidence.

A further type of bias associated with the adoption of AI in radiology is the automation bias. It consists of preferring automated responses despite the presence of conflicting information, effectively deferring decision-making competence to the automated system [5,12,37]. This phenomenon occurs when radiologists, especially those with less experience, overrely on AI systems, assuming that the absence of findings meant there was no abnormality, which can lead to missed diagnoses [4].

In a related vein, the satisfaction of report bias refers to the tendency of a radiologist to perpetuate radiological error that occurred in a previous assessment [5,39]. A radiologist could tend to accept a prior report or AI finding as sufficient, which prematurely halts the search for additional abnormalities. As a result, a second radiologist may be influenced by the judgment of a first radiologist or by AI tools, perpetuating mistakes. This does not mean that comparison with previous exams [41] or the use of AI systems should be avoided, as it can improve diagnostic accuracy. To mitigate these different types of errors, radiologists should always evaluate images independently before consulting past reports or AI interpretation outcomes.

One prominent cognitive bias is the attribution bias. It occurs when the characteristics of the patients, often shaped by stereotypes, inappropriately influence the radiologist’s diagnostic reasoning process, causing them to neglect other peculiar and relevant information [5,35,39]. AI has the potential to reduce this bias by focusing solely on current imaging data, without being influenced by the patient’s medical history or previous clinical context, thereby helping radiologists avoid inappropriate assumptions and improve diagnostic accuracy. Another possible bias is the framing bias that highlights the tendency to interpret a diagnosis according to how the clinical scenario is presented to the radiologist, leading them to misjudge the significance of the findings [5,39,42]. A specific symptom or diagnostic imaging finding located within one anatomical-physiological system may potentially divert the radiologist’s attention from the involvement of an alternative system [43]. In this case, the dataset on which the AI is trained must be optimal [36]. If the AI is trained on clinical datasets that contain inherent biases or distorted presentations, it could inadvertently reinforce framing bias by reflecting the same distortions in its analyses and recommendations.

Furthermore, the satisfaction of search bias occurs when a radiologist ceases a visual search for additional clinically significant abnormalities upon the initial pathology being identified [5,35,43,44]. This can lead to missed diagnoses of additional issues that may also be present. If the radiologist merely responds to the attending physician’s question, other important findings may be overlooked if the radiologist does not perform a comprehensive assessment [43]. AI tools can counteract this phenomenon by prompting comprehensive image reviews, flagging secondary findings, and encouraging comprehensive assessment.

Similarly, some symptoms or entirely visible and clinically significant findings can be neglected because one’s attention is fixed on another task or object (ie, inattentional blindness bias) [35,39]. Compelling evidence for this phenomenon was observed in a study conducted in the radiological field, where 24 radiologists were asked to detect lung nodules. In the last CT scan, a gorilla 48 times larger than the average nodule was inserted. About 83% of radiologists failed in their recognition [45,46]. This error occurred not because the lesion was subtle or invisible, but because attention was narrowly directed to the suspected finding, leading to inattentional blindness: the failure to see a visible but unexpected object when attention is engaged elsewhere. This type of bias could be reduced by AI systems that objectively highlight all anomalies detected in the image, drawing attention to findings that radiologists might overlook due to the limitations of cognitive concentration.

The phenomenon of hindsight also plays a crucial role in radiological errors, as radiologists may underestimate the complexity of reaching the initial diagnosis and overestimate the probability of a diagnosis based on prior knowledge of the outcome [5,35,43].

The radiologist’s judgment during the review is influenced by hindsight bias, the tendency to see events as more predictable or obvious after they have already occurred. AI, relying on algorithms and statistical models rather than retrospective knowledge, provides consistent and unbiased analysis that is not influenced by previous results, potentially protecting against this bias.

Conversely, regret bias leads to the overreporting of certain diseases due to the negative consequences associated with missed diagnoses [5,43]. Similar to the previous bias, AI can mitigate this emotional and experience-based bias by offering probabilistic assessments based on large datasets, helping physicians strike a balance between caution and evidence-based reasoning.

Another bias to which breast radiologists are commonly exposed is the commission bias, representing the urge to do something that might be unnecessary, such as recalling a patient, ordering additional imaging, or recommending a biopsy, instead of doing nothing [43]. The commission bias might happen in different contexts, but breast imaging is very vulnerable to this bias due to the high psychological, clinical, and medicolegal pressures of this field. Nevertheless, this approach might lead to avoidable expenses and increase the likelihood of false-positive findings, which may prompt unwarranted biopsies or additional imaging, ultimately outweighing any perceived benefit for these patients [47]. AI can mitigate commission bias by acting as an objective second reader, providing quantitative malignancy probability scores to challenge subjective overcalls on benign asymmetries.

Finally, premature closure bias arises when a radiologist prematurely accepts a diagnosis as definitive before any alternative diagnoses are considered [5,35,39]. When trained with adequate datasets [36], AI can assist by suggesting alternative diagnoses based on image characteristics and differential analyses, encouraging radiologists to maintain an open diagnostic approach and avoid hasty conclusions.

In this scenario, the concept of algorithm aversion has also become increasingly relevant. This term refers to the psychological tendency of individuals to distrust and avoid algorithmic decision support, especially after observing even minor errors, despite evidence that algorithms often outperform human judgment. Foundational work by Dietvorst and colleagues [48] demonstrated that when individuals witness predictive algorithms making mistakes, they subsequently prefer human forecasters. Building on this, Dietvorst and Bharti [49] demonstrated that a diminishing sensitivity to forecasting error further reinforces algorithm rejection in uncertain decision-making domains, as people overweight early errors and underweight later accuracy improvements.

Similar dynamics can be observed in breast radiology, where radiologists’ interactions with diagnostic AI systems critically shape trust and subsequent use. Early AI misclassifications can disproportionately reduce reliance on algorithmic output, particularly among clinicians with high diagnostic self-efficacy [50]. These patterns of algorithm aversion may hinder the integration of AI tools into routine clinical practice. Consequently, radiologists may continue to favor their own judgments despite well-known human limitations such as fatigue, perceptual bias, and interreader variability. In breast imaging specifically, algorithm aversion may lead to missed opportunities for AI-assisted early cancer detection, reduced returns on investment in AI technologies, and slower adoption of tools capable of improving population-level outcomes.

## Preventive Strategies and Future Directions

Preventive strategies aimed at reducing potential physicians’ errors represent a critical topic and should be developed based on the most common errors encountered in clinical practice [6].

A fundamental consideration is providing comprehensive education on AI, increasing AI literacy, and enabling individuals to make informed choices [4]. An appropriate education allows health care professionals to be equipped with awareness of its risks and benefits, along with the necessary skills to identify, prevent, and address potential failures arising from its application in radiology [4]. Specifically, targeted training programs designed to reduce certain cognitive errors are essential [51]. For instance, Taussig and colleagues [52] proposed an innovative approach where senior residents present selected interpretative errors to junior residents, fostering peer discussion that enhances knowledge and helps prevent the recurrence of errors.

Continuing education should also foster insight and metacognitive skills by including structured explanations of cognitive biases and clinical examples illustrating their effect on diagnostic reasoning [51]. Encouraging clinicians to critically reflect on their diagnostic processes, rather than relying on immediate problem-solving, can improve diagnostic accuracy. Simulation techniques, such as cognitive exercises and mental rehearsals, further support this learning by exposing trainees to the impacts of biased versus unbiased reasoning [51]. Additionally, training videos and case comparisons reinforce debiasing strategies and facilitate the transfer of these skills to everyday practice.

Another effective strategy to reduce the frequency of cognitive bias in radiological report interpretation is the establishment of a clear communication strategy among health care professionals. Indeed, ineffective communication due to inefficient team interaction or interprofessional tension increases the risk of making medical errors [53]. The European Society of Radiology published in 2013 communication guidelines to provide helpful information on conducting an effective discussion between patients, referrers, colleagues, and students [54]. Furthermore, improving communication between clinicians and radiologists promotes their collaboration, which, in turn, might help in preventing cognitive errors such as attribution, anchoring, or confirmation bias caused by the lack of knowledge about the patient’s medical history and proper clinical context, with subsequent incorrect interpretation of the images. Effective communication and discussion with colleagues also allow for timely feedback regarding the clinical decision and enable an understanding of the error and implementation of strategies to correct it [51].

Beyond education and communication, bias mitigation should also be addressed at a structural level through workflow engineering and explainable artificial intelligence (XAI).

Integrating AI into clinical engineering workflow in medical imaging entails structured interpretation sequences through strategic design of reading protocols to optimize diagnostic accuracy while counteracting cognitive biases [55,56]. Sequential workflows require radiologists to interpret images independently before consulting AI-generated marks in a second-reader paradigm, enhancing sensitivity but potentially reducing specificity due to increased false positives and extended reading times [57]. It preserves independent judgment to prevent bias, such as premature closure.

Concurrent integration, by contrast, offers real-time validation against heuristics like anchoring on salient features. Concurrent reading integrates AI annotations during initial interpretation, improving both sensitivity and specificity while reducing reading time [57].

Furthermore, XAI approaches can foster calibrated trust by enabling clinicians to interrogate the rationale and uncertainty of AI outputs rather than passively following algorithmic recommendations. XAI encompasses methods that make deep learning models intelligible to human users, clarifying their internal logic, decision pathways, and case-specific predictions, as well as their capabilities and limitations, instead of treating them as opaque black boxes [58]. In medical imaging, XAI does not remove bias but supports its detection, interpretation, and mitigation by revealing model decision rules and the data patterns on which they rely. By providing interpretable feature attributions, XAI helps counter cognitive biases such as the availability heuristic and anchoring, allowing clinicians to cross-check AI suggestions against the actual imaging findings and to avoid overreliance on salient anomalies [32]. These techniques jointly target algorithmic biases originating from datasets, training procedures, and generalization failures, as well as clinicians’ own cognitive biases, thereby promoting a more critical and informed use of algorithmic outputs and enhancing diagnostic accuracy while preserving transparency, including for underrepresented patient groups in training data [32,58].

Integrating AI into radiological practice enhances diagnostic workflows and operational efficiency by reducing workload and optimizing time, critical factors in preventing burnout and fatigue-related errors [56]. The study conducted by Bruno and collaborators explored possible approaches to minimize errors in the field of radiology, including a reduction of working hours, a minimization of interruptions and distractions, and an alleviation of the pressure to maintain a rapid workflow [59]. However, such interventions face practical challenges in some hospital settings and have shown minimal impact. Systematic studies are needed to evaluate how AI can be effectively incorporated into image interpretation processes to support accurate and efficient everyday clinical practice.

## Conclusion

The integration of AI in breast imaging represents a promising advancement in radiology, with the potential to improve diagnostic accuracy, optimize workflow, and reduce perceptual, attentional, and interpretive errors. However, as this perspective highlights, AI is not a flawless solution and introduces new challenges, including cognitive biases that may influence radiologists’ decision-making. Understanding these biases is crucial to implementing strategies to mitigate these errors, ensuring that AI serves as an effective aid rather than a source of further diagnostic problems.

While AI offers substantial advantages, a cautious approach is required to mitigate its potential risks and limitations. To fully exploit the benefits of AI while minimizing its limitations, further research should focus on optimizing the AI integration into clinical workflows, assessing its impact on diagnostic reasoning, and developing structured guidelines to mitigate automation biases and other cognitive distortions. A successful AI implementation in breast imaging will ultimately require a synergistic approach that blends human expertise with technological innovation. A balanced approach harnessing AI as a complementary tool while maintaining radiologists’ critical thinking and expertise will improve breast cancer diagnosis.

## Supplementary material

10.2196/78955 Multimedia Appendix 1 Literature search.

## Abbreviations

AI: artificial intelligence
CAD: computer-aided detection
CT: computed tomography
DBT: digital breast tomosynthesis
MASAI: mammography screening with artificial intelligence
XAI: explainable artificial intelligence
