# Supplementary material for: Viewpoint on the Consequences and Mitigation of Cognitive Bias in the Radiological Interpretation of Breast Cancer Imaging Using Artificial Intelligence
Source: JMIR Med Inform. 2026 Mar 30;14:e78955. doi: 10.2196/78955 (PMC13035202; doi:10.2196/78955)
Supplement: Multimedia Appendix 1 [file medinform-v14-e78955-s001.docx]

**Literature search**

A comprehensive literature search was conducted in PubMed, Embase, and Google Scholar using combinations of the keywords “Radiological assessment,” “Artificial Intelligence,” “Cognitive bias,” and “Breast cancer imaging”, up to December 2025. The search focuses mainly on the publications from January 2013 to December 2025 to capture the most recent developments in the field. Eligible sources included peer‑reviewed original research articles, systematic reviews, and meta‑analyses focusing on the clinical implementation of AI in breast cancer imaging, workflow integration, interpretative performance, and cognitive bias. Studies were excluded if they were not written in English, lacked clinical applicability, or focused solely on technical model development without clinical evaluation.
